# Transforming Spent Coffee Grounds’ Hydrolysates with Yeast *Lachancea thermotolerans* and Lactic Acid Bacterium *Lactiplantibacillus plantarum* to Develop Potential Novel Alcoholic Beverages

**DOI:** 10.3390/foods12061161

**Published:** 2023-03-09

**Authors:** Yunjiao Liu, Yuyun Lu, Shao-Quan Liu

**Affiliations:** 1 Department of Food Science and Technology, Science Drive 2, Faculty of Science, National University of Singapore, Singapore 117542, Singapore; 2 National University of Singapore (Suzhou) Research Institute, 377 Lin Quan Street, Suzhou Industrial Park, Suzhou 215123, China

**Keywords:** non-*Saccharomyces* yeast, lactic acid bacteria, co-inoculation, sequential inoculation, spent coffee grounds

## Abstract

In the present work, the modification of spent coffee grounds (SCG) hydrolysate composition by mixed cultures of a non-*Saccharomyces* yeast, *Lachancea thermotolerans*, and a lactic acid bacterium, *Lactiplantibacillus plantarum*, as well as their interactions, were evaluated. It was found that *L. plantarum* inhibited the growth and survival of *L. thermotolerans* as compared with that in the yeast alone. On the other hand, the growth and survival of *L. plantarum* was slowed in sequential fermentation, but not in co-culture. Compared with co-culture, higher ethanol content, less residual sugars, and less acetic and succinic acids were found in sequential fermentation. In addition, lower amounts of caffeine and phenolic acids (e.g., ferulic, caffeic, and p-coumaric acids) were obtained in mixed (co- and sequential) cultures with corresponding levels of volatile phenols relative to the yeast monoculture. Moreover, co-culturing resulted in the highest contents of total alcohols (ethanol excluded) and total esters. Therefore, mixed culturing of *L. plantarum* and *L. thermotolerans* presented positive effects on the chemical constituents of fermented SCG hydrolysates, which might be a new alternative approach to valorizing the SCG into novel alcoholic drinks with different ethanol and flavor constituents.

## 1. Introduction

It is well known that yeasts play a vital role in the chemical composition and quality of wine and other alcoholic beverages [1,2]. Generally, *Saccharomyces* yeasts conduct alcoholic fermentation (AF) via the biotransformation of fermentable sugars (e.g., fructose and glucose) to ethanol and other chemicals (e.g., organic acids, glycerol, acetaldehyde, and esters) affecting the taste and aroma of the final product [3,4]. Since *Saccharomyces* yeasts have been extensively investigated, the influence of non-*Saccharomyces* yeasts on AF attracts more and more attention [2,4]. It has been reported that some non-*Saccharomyces* yeasts (e.g., *Lachancea thermotolerans* and *Torulaspora delbrueckii*) exhibit a comparable ethanol production capacity to *Saccharomyces* yeasts when used in some fruit wines fermentation such as durian [5,6], and also produce increased levels of higher alcohols, esters, and carbonyl compounds [7].

To enhance the quality of wine and other alcoholic beverages, malolactic fermentation (MLF) is sometimes conducted together with AF to modify the aroma and taste, stabilize from a microbiological point of view, and/or deacidify the final product via the biotransformation of malate to lactate and CO_2_ by lactic acid bacteria (LAB) [8,9]. For MLF, *Oenococcus oeni* is the most widely used LAB species due to its good capability of catabolizing malic acid, growing well in various media, and generating pleasant aroma compounds (e.g., diacetyl, esters, and higher alcohols). However, this kind of LAB can produce undesirable levels of acetic acid from excess sugars if it grows spontaneously or is inoculated early in the yeast fermentation stage when the sugar level is still high [10].

Recently, another LAB strain, *Lactiplantibacillus plantarum* (formerly *Lactobacillus plantarum*) was introduced to perform MLF. It was found that *L. plantarum* strains can survive and grow well under harsh environmental wine conditions (e.g., lower pH values of pH 3.5 or above and higher ethanol contents up to 14%) [8,11,12,13]. In addition, these strains can consume malic acid as well as some sugars and decrease volatile acidity due to their facultative heterofermentative (i.e., homofermentative) character such that they do not produce acetic acid from hexoses, although they produce acetic acid from pentoses that are usually present at low levels [11,12,13]. Furthermore, it has also been reported that *L. plantarum* strains produce a broader diverse enzymatic profile than that of *O. oeni* strains and that these extracellular enzymes play important roles in modifying wine aroma compositions [8].

Previous studies have explored the combination of some non-*Saccharomyces* species (e.g., *Starmerella bacillaris* and *Hanseniospora uvarum*) and *L. plantarum* [3,13,14]. A previous study found that the co-inoculation of *H. uvarum* and *L. plantarum* not only improved the aroma and sensory properties of the wine, but also shortened the time for MLF [3]. Interestingly, the combination of *C. zemplinina* and *L. plantarum* may stimulate, inhibit, or not affect the MLF depending on the inoculation regime and the strain used [13,14]. In addition, Urbina and co-authors investigated the combination of *L. thermotolerans* and *L. plantarum* in wine technology and they found that the co-inoculation improved the aroma property [15]. These studies indicate that it is very important to investigate the compatibility between different yeast and bacterial species/strains.

Spent coffee grounds (SCG), the solid food waste after coffee brewing, has been reused as a fermentable substrate for producing experimental alcoholic beverages (ethanol content < 10%, *v*/*v*) [4,9,16,17]. For example, in our previous studies, mono-inoculation of *Saccharomyces* yeasts such as *S. cerevisiae* MERIT and non-*Saccharomyces* yeasts such as *Pichia kluyveri*, *T. delbrueckii*, and *L. thermotolerans* have been investigated in SCG hydrolysates [4,9]. In addition, the simultaneous AF with *L. thermotolerans* and MLF with three *O. oeni* strains (PN4, Enoferm Beta, and Lalvin 31) in SCG hydrolysates were also investigated, while a stuck fermentation was observed when co-inoculated with *O. oeni* Enoferm Beta and *O. oeni* PN4, which was attributed to the early yeast cell death [16]. The early yeast cell death was ascribed to a lack of survival factors or certain nutrients (consumption by bacteria), or due to the higher production of acetic acid by bacteria. Therefore, other alternative inoculation strategies such as sequential inoculation or replaceable LAB strains may mitigate or avoid the above-mentioned problems. To the best of our knowledge, there is no report on the combination of *L. thermotolerans* and *L. plantarum* in SCG hydrolysates yet.

Hence, we aimed to investigate the impacts of *L. plantarum* in mixed fermentation with *L. thermotolerans* via different inoculation strategies (mono-, co- and sequential inoculations) on the non-volatile and volatile components of SCG hydrolysates. Microbial growth kinetics, changes in sugars, organic acids, phenolics, and alkaloids, as well as volatile compounds, were monitored. The changes in antioxidant capacity of SCG hydrolysates were also measured before and after fermentation.

## 2. Materials and Methods

### 2.1. SCG Hydrolysates Preparation

One hundred fifty grams of defatted and dried SCG (oven dry, moisture content < 5%, *w*/*w*) was ground into fine powder and suspended with deionized water to form a volume of 1 L of an aqueous mixture [4,9]. The SCG hydrolysates were obtained by sequential acidic and enzymatic hydrolysis of one liter of SCG aqueous mixture with 0.2 M citric acid at 121 °C for 1 h and with 6% Viscozyme^®^L (*v*/*w*, a cellulolytic and hemicellulolytic enzyme mixture) at 50 °C for 24 h, respectively [4].

Prior to fermentation, the SCG hydrolysates were supplemented with sucrose to reach a °Brix value of 15 (pH = 5) to enrich the carbon sources and yeast extracts (0.25%, *w/v*) to enrich the nitrogen sources. The supplemented SCG hydrolysates were pasteurized (60 °C, 30 min), and the pasteurization efficiency (no viable cells on plates) was verified by two types of spread plating on (type 1) potato dextrose agar (PDA) (Basingstoke, Hampshire, England) for yeasts and (type 2) MRS agar (Merck, Singapore) for bacteria as described previously [9].

### 2.2. Inoculum Preparation

*L. thermotolerans* Concerto (Chr. Hansen, Horsholm, Denmark) was purchased in freeze-dried form. The freeze-dried yeast powder was activated and propagated in 100 mL of sterile yeast nutrient broth (pH 5.0) containing 0.25 g of yeast extracts, 0.25 g of bacteriological peptone, 0.25 g of malt extracts, and 2 g of glucose. The yeast culture was incubated (20 °C, 72 h), collected, and kept at −80 °C (30% glycerol was added before freezing) before use. The freeze-dried *L. plantarum* ML Prime (Lallemand Inc., Brooklyn Park, Edwardstown, Australia) was activated and incubated in a modified sterilized MRS broth consisting of 80 mL of MRS broth (Merk, Singapore, Singapore) and 20 mL of apple juice (pH 5.5, Marigold, Malaysia Dairy Pte. Ltd., Singapore, Singapore) at 25 °C for 96 h. The pure culture of *L. plantarum* ML Prime was collected and kept at −80 °C (mixed with 30% *v*/*v* sterile glycerol) before use.

### 2.3. Microbial Fermentation Design

Pure cultures of *L. thermotolerans* Concerto and *L. plantarum* ML Prime were firstly reactivated in yeast nutrient broth (20 °C, 3 days) and modified MRS broth (25 °C, 4 days) as described above, respectively. The precultures of *L. thermotolerans* and *L. plantarum* were prepared separately by pipetting respective pure culture (5%, *v*/*v*) into 100 mL of modified SCG hydrolysates, which were incubated at 20 °C for 3 days for yeast and at 25 °C for 4 days for bacteria with cells grown to at least 7 log CFU/mL.

For mono-inoculations, precultures of *L. thermotolerans* (~10^7^ CFU/mL, 1%, *v*/*v*,) and *L. plantarum* ML Prime (~10^8^ CFU/mL, 1%, *v/v*) were separately pipetted into corresponding 300 mL of pasteurized SCG hydrolysates, defined as LT and LP. The co-inoculation of *L. thermotolerans* (1%, *v*/*v*) and *L. plantarum* ML Prime (1%, *v*/*v*) was performed and defined as LT+Co-LP. The sequential inoculation was conducted via inoculating 1% (*v*/*v*) of *L. plantarum* ML Prime preculture into SCG hydrolysates pre-fermented with *L. thermotolerans* at day 4 and defined as LT+Se-LP. All treatments were statically fermented at 25 °C for 14 days. Samples were collected periodically and kept at −20 °C before analysis. Cell enumeration was conducted by spread plating with PDA plates (containing 0.1 g/L chloramphenicol) for *L. thermotolerans* Concerto and MRS plates (containing 0.1 g/L Natamax) for *L. plantarum* ML Prime.

### 2.4. Total Soluble Solids Contents and pH Measurements

The measurements of total soluble solids contents and pH were performed by using an RX-5000a refractometer (ATAGO, Tokyo, Japan) and a pH meter (Metrohm, Herisau, Switzerland), respectively.

### 2.5. Analysis of Non-Volatile Composition

Sugars and glycerol were analyzed by using a Shimadzu HPLC (Kyoto, Japan) and a Zorbax carbohydrate column (150 × 4.6 mm, Agilent, Santa Clara, CA, USA) connected to an evaporative light scattering detector (ELSD) with 80% acetonitrile as a mobile phase [4]. Ethanol, organic acids, phenolic acids, and alkaloids were quantified by a Shimadzu HPLC (Kyoto, Japan), detailed in our previous study [4]. Phenolic acids and alkaloids were measured by HPLC connected with a Zorbax Eclipse C18 column under gradient elution with mobile phase A (0.1% *v*/*v* acetic acid in H_2_O) and mobile phase B (100% methanol) and connected with a PDA detector at 320 nm. Free amino compounds were analyzed by ARACUS Amio Acid Analyzer (MembraPure, Berlin, Germany) under a hydrolysate separation program [4].

### 2.6. Analysis of Volatile Composition

Volatile profiles were detected by following a previous study [9], where 5 mL of each sample (final pH 2.5, adjusted with 1 M HCl) in a 20 mL GC vial was extracted by headspace solid-phase microextraction (HS-SPME) and a carboxen/polydimethysiloxane fiber (85 μm; Supelco, PA, USA). The extracted volatile compounds were further separated on a capillary column (DB-FFAP, 60 m x 0.25 mm, 0.25 mm, i.d.) with helium (1.2 mL/min) and detected by an Agilent GC-MS/FID (Santa Clara, CA, USA). The identification of volatiles was performed by comparison with NIST 14 library and further verified by the linear retention index (LRI) values from C10-C40 alkanes.

### 2.7. Antioxidant Assays

2,2-Diphenyl-1-picrylhydrazyl (DPPH) and oxygen radical absorbance capacity (ORAC) assays were conducted by following reported methods [18] to measure the antioxidant capacity of unfermented and fermented SCG hydrolysates. The antioxidant capacity was expressed as microgram of Trolox equivalents per liter of sample (μg TE/L) for DPPH assay and mmol of TE per liter of sample (mmol TE/L) for ORAC assay.

### 2.8. Statistical Analysis

All data from independent triplicate fermentations (*n* = 3) were recorded as the average value ± standard deviation (SD). One-way ANOVA with Tukey’s post hoc test was conducted by SPSS^®^ 20.0 (Windows version, Chicago, IL, USA) to check the significant differences (*p* < 0.05) among samples. Heatmap was plotted by Heatmapper [19]. Principal component analysis (PCA) of selected aromatic compounds was performed by Origin 2021b (OriginLab Corporation, Northampton, MA, USA).

## 3. Results and Discussion

### 3.1. Microbial Growth

**Figure 1:**
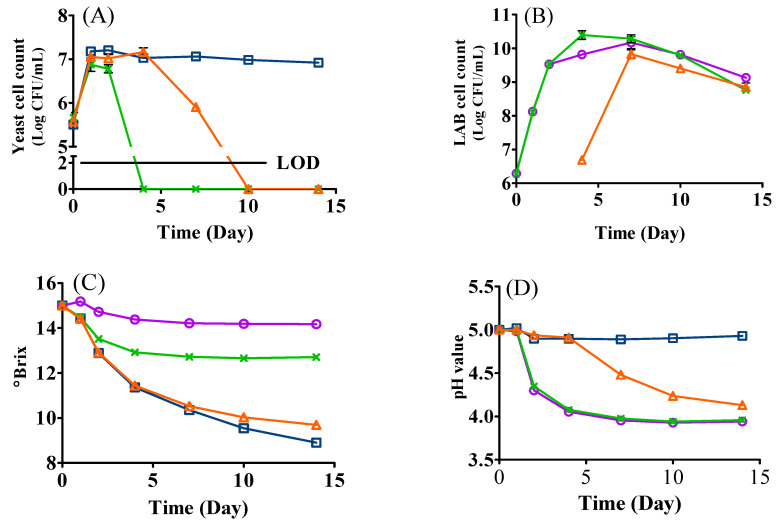
Changes in cell counts of *L. thermotolerans* Concerto (**A**), *L. plantarum* ML Prime (**B**), °Brix (**C**), and pH (**D**) during the fermentation of SCG hydrolysates. (□) LT: mono-inoculation of *L. thermotolerans* Concerto; (○) LP: mono-inoculation of *L. plantarum* ML Prime; (X) LT+Co-LP: co-inoculation of *L. thermotolerans* Concerto and *L. plantarum* ML Prime; (△) LT+Se-LP: sequential inoculation of *L. thermotolerans* Concerto and *L. plantarum* ML Prime at day 4. LOD, limit of detection for yeast was 2 log CFU/mL. LOD, limit of detection.

The microbial growth kinetics of the mono- and mixed-culture fermentations inoculated with *L. thermotolerans* Concerto and *L. plantarum* ML Prime are shown in Figure 1A,B. In general, the growth of yeast and LAB presented different trends. For the yeast, it was significantly adversely affected by the introduction of *L. plantarum* ML Prime. For example, in co-inoculation, although the yeast grew well on day one, the cell count (6.87 Log CFU/mL) was relatively lower than that of mono-inoculation (LT, 7.18 Log CFU/mL) (Figure 1A); after that, the yeast cell count in co-inoculation decreased sharply and was not detected from day four. The yeast cell count in sequential inoculation followed a similar growth trend to yeast mono-inoculation during the first 4 days’ fermentation. Once *L. plantarum* ML Prime was inoculated on day four, the yeast count declined remarkably and could not be detected on day seven in the sequential inoculation (Figure 1A). In contrast, the cell count in yeast mono-inoculation stayed relatively stable until day 14 (Figure 1A). Our results agreed with Bartle and coauthors [20], who also reported the inhibitory effects of LAB on the yeast growth in mixed cultures. The early death of *L. thermotolerans* in mixed inoculations might be ascribed to the production of a variety of antifungal substances (e.g., palmitic acid, 2-butyl-4-hexyloctahydro-1H-indene, and 3-phenyllactic acid) by *L. plantarum* [21].

For *L. plantarum* ML Prime (Figure 1B), the cell count in the mono-inoculation increased from 6.28 Log CFU/mL (day zero) to 10.17 Log CFU/mL on day seven, while the LAB cell count increased to the highest population of 10.39 Log CFU/mL on day four in co-inoculation and 9.82 Log CFU/mL on day seven in sequential inoculation. (Figure 1B). This indicated that, although yeast died early, the ethanol likely inhibited the LAB, supported by the different ethanol levels and different LAB counts in co- and sequential fermentation. The results agreed with a previous study [10], where the highest cell population of LAB in mixed inoculation was also similar to that of LAB mono-inoculation. In addition, the decline of LAB may also be due to the lack of nutrients or the accumulation of other substances such as medium-chain fatty acids and certain peptides/proteins [9,10].

### 3.2. Changes in °Brix and pH

The changes of °Brix are shown in Figure 1C. The °Brix value in yeast mono- and sequential inoculation continuously decreased to 8.90 and 9.69, respectively (Table 1). In co-inoculation, the °Brix value slightly decreased to 12.92 during the first 4 days’ fermentation and then remained stable (Figure 1C). This could be ascribed to the early death of the yeast in co-inoculation (Figure 1A). The relatively stable °Brix value in LAB mono-inoculation was expected because *L. plantarum* ML Prime may consume less fermentable sugars than yeasts [15].

The changes of pH indicated the variance of acidity of the media as presented in Table 1 and Figure 1D. In LAB mono- and co-inoculated samples, the pH value (pH 5.00) decreased from day one to day seven and then stayed stable at 3.94 and 3.95, respectively. However, in sequential inoculation, the pH continuously decreased until day 14 to pH 4.13 once *L. plantarum* ML Prime was inoculated, while in yeast mono-inoculation, the pH value remained relatively stable during the 14-day fermentation (Figure 1D).

### 3.3. Changes in Sugars

**Figure 2:**
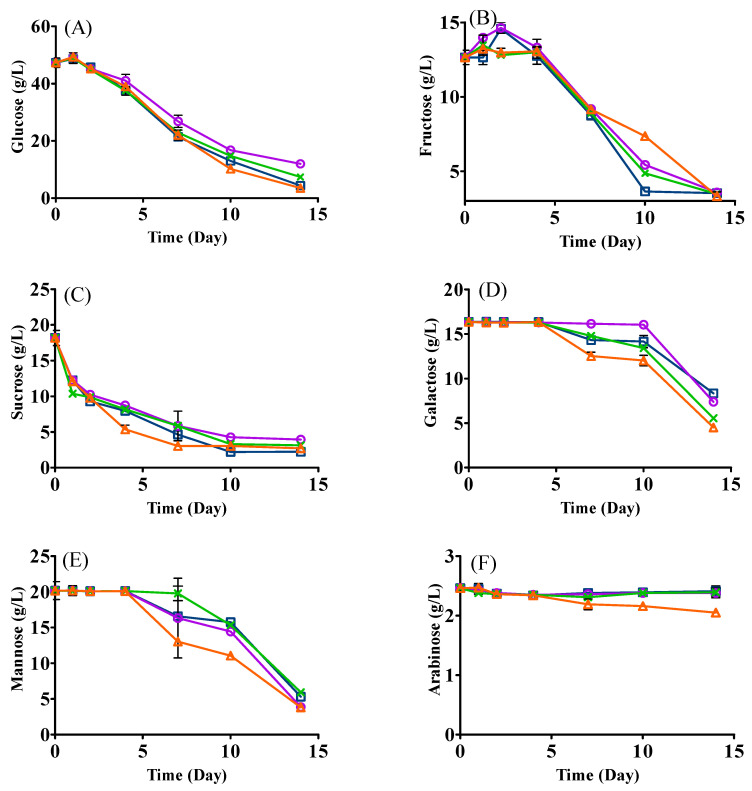
Changes in sugars ((**A**), glucose; (**B**), fructose; (**C**), sucrose; (**D**), galactose; (**E**), mannose; (**F**), arabinose) in SCG hydrolysates fermented with *L. thermotolerans* and *L. plantarum*. LT (□): mono-inoculation of *L. thermotolerans* Concerto; LP (○): mono-inoculation of *L. plantarum* ML Prime; LT+Co-LP (X): co-inoculation of *L. thermotolerans* Concerto and *L. plantarum* ML Prime; LT+Se-LP (△): sequential inoculation of *L. thermotolerans* Concerto and *L. plantarum* ML Prime at day 4.

The changes of sugars are shown in Figure 2. In general, all sugars decreased to different extents after fermentation (Figure 2). The consumption of glucose and sucrose showed similar trends in all treatments with small residual amounts (fructose: 3.33–3.58 g/L; sucrose: 2.23–3.13 g/L) on day 14 (Figure 2A,C). Glucose significantly decreased in yeast mono- and sequential inoculation with the residual levels of 3.53 g/L and 4.40 g/L, respectively. Interestingly, there was no significant decline of galactose and mannose for all treatments during the first 4 days’ fermentation, which might be due to sucrose, glucose, and fructose being the most preferred carbohydrates. After day four, sequential and co-inoculation metabolized more galactose as compared with the mono-inoculation of yeast or LAB, which could be due to the lack of main carbohydrates (e.g., glucose and fructose) and a good capacity of *L. plantarum* ML Prime to catabolize galactose to support its growth and metabolism [22]. Differently, sequential and LAB mono-inoculation consumed more mannose on day 7 and day 10 as compared with the yeast mono-inoculation, possibly as a result of *L. plantarum* ML Prime catabolizing mannose by its mannose phosphotransferase system [23]. For arabinose, it generally remained relatively stable in all fermentations, although it declined slightly in sequential inoculation (Figure 2F). The results agreed with our previous studies [4,9], where both yeasts and LAB did not consume arabinose.

### 3.4. Changes in Organic Acids

**Figure 3:**
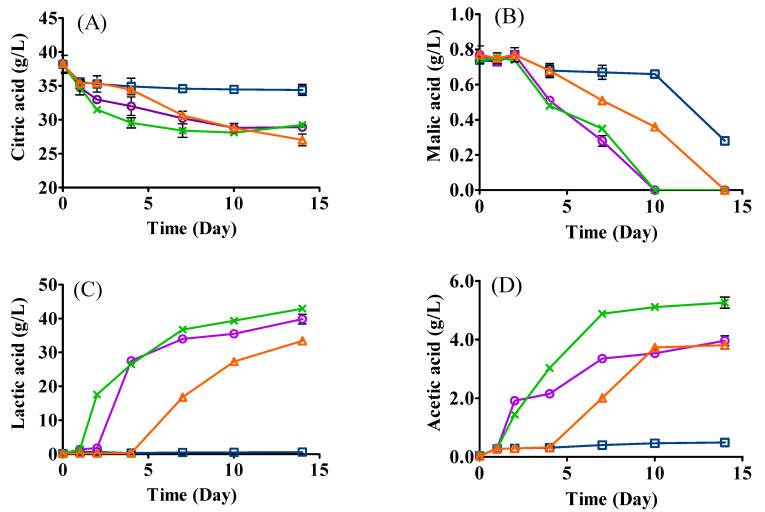

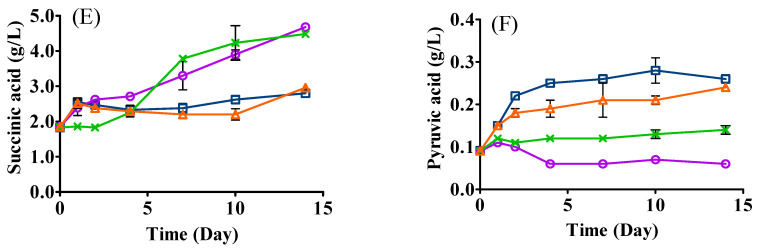
Changes in organic acids ((**A**), citric acid; (**B**), malic acid; (**C**), lactic acid; (**D**), acetic acid; (**E**), succinic acid; (**F**), pyruvic acid) in the fermentation of SCG hydrolysates. LT (□): mono-inoculation of *L. thermotolerans* Concerto; LP (○): mono-inoculation of *L. plantarum* ML Prime; LT+Co-LP (X): co-inoculation of *L. thermotolerans* Concerto and *L. plantarum* ML Prime; LT+Se-LP (△): sequential inoculation of *L. thermotolerans* Concerto and *L. plantarum* ML Prime at day 4.

Significant decreases in citric acid and malic acid were observed in all samples, especially in the samples inoculated with *L. plantarum* ML Prime (Figure 3A,B). The high initial amount of citric acid (38.16 g/L) was carried over by the acidic hydrolysis of SCG. It has been reported that *L. plantarum* ML Prime shows an excellent ability to metabolize citric acid to lactic acid [24]. Indeed, a significantly higher production of lactic acid was detected in samples inoculated with *L. plantarum* ML Prime, namely mono- (39.84 g/L), co- (42.92 g/L), and sequential inoculation (33.41 g/L), as compared with that in yeast mono-inoculation (LT, 0.58 g/L) (Figure 3C). The high production of lactic acid could be due to the high metabolism of citrate to lactate [25] and the conversion of sugars to lactic acid by *L. plantarum* ML Prime [26]. The lower level of lactic acid in sequential inoculation than that of co-inoculation could be ascribed to the shorter fermentation period, prior consumption of sugars by yeast and lower cell population of LAB in the sequential fermentation.

For malic acid, it could not be detected on day 10 for LAB mono- and co- inoculation and on day 14 for sequential inoculation (Figure 3B). The decrease in malic acid could mainly be ascribed to the metabolism to lactic acid by *L. plantarum* ML Prime via malolactic enzyme [25,27].

The highest concentration of acetic acid was detected in co-inoculation (5.26 g/L), followed by LAB mono-inoculation (3.96 g/L), sequential inoculation (3.81 g/L), and yeast mono-inoculation (0.49 g/L). It is clear that fermentations involving *L. plantarum* ML Prime produced significantly higher amounts of acetic acid (Figure 3D), which correlated to the higher citric acid consumption by this LAB. Citric acid can be catabolized to acetic acid, lactic acid, and other minor metabolites such as acetoin [24]. The higher amount of acetic acid produced in co-inoculation could be produced from amino acids such as serine by *L. plantarum* [28]. Less acetic acid produced in sequential inoculation would be beneficial for the final aroma and flavor of the fermented SCG hydrolysates, since excess acetic acid could induce a vinegary flavor [29].

Similar amounts of succinic acid were produced in LAB mono- (4.68 g/L) and co-inoculation (4.48 g/L) (Figure 3E), while a significantly lower amount was produced in sequential inoculation (2.96 g/L). Citric acid is known to be converted to succinic acid by some LAB via the reduction sequence of the TCA cycle [30]. The lower amount of succinic acid produced in sequential inoculation might be due to less citric acid being converted to succinic acid by *L. plantarum* ML Prime [25]. The metabolic interactions between yeast and LAB may also differ in co- and sequential inoculations, affecting succinic acid formation. Our results were in line with Martín-García and coauthors [31], who also reported that the sequential inoculation of yeast (*T. delbrueckii* Biodiva) and LAB (*O. oeni* PSU-1) produced a lower content of organic acids (e.g., L-malic acid and succinic acid) than that in simultaneous inoculation in white grape wine.

Small amounts of pyruvic acid were generated in yeast mono- (0.26 g/L) and sequential inoculation (0.24 g/L). The significantly lower concentration of pyruvic acid in co-inoculation (0.14 g/L) could be ascribed to the early yeast cell death, which affected the glycolysis metabolism [4], and/or consumption of pyruvic acid by *L. pantarum*.

### 3.5. Changes in Alkaloids, Phenolic Acids, and Antioxidant Capacity

Alkaloids (trigonelline, caffeine, theobromine, and theophylline) and phenolic acids (caffeic, chlorogenic, *p*-coumaric, and ferulic acids) were quantified in all samples (Table 2). Interestingly, trigonelline significantly declined in co-inoculation, while in LAB mono- and sequential inoculation, there was a decreasing trend (Table 2). It has been reported that trigonelline might be converted to nicotinic acid by yeasts [32], but it is unknown whether *L. plantarum* ML Prime could exhibit the same ability. Theobromine decreased significantly in all samples, especially in yeast mono- and sequential inoculation (Table 2). Our results followed a similar trend to the SCG hydrolysate co-fermented with *L. thermotolerans* Concerto and *O. oeni* [9], and the reduction might be because theobromine was degraded to xanthine as reported in fungi (e.g., *Penicillium commune*) [33]. Theophylline showed different changes among different treatments; for example, it significantly declined in the yeast mono-inoculation, stayed relatively stable in LAB mono- and co-inoculation, but significantly increased in the sequential inoculation (Table 2). The varied changes of theophylline may be due to the enzymatic activity of *L. plantarum* and its different interaction with *L. thermotolerans* among treatments [25], while the specific LAB’s enzyme activity requires further exploration. Caffeine significantly decreased in samples involving *L. plantarum* ML Prime, suggesting that caffeine might have been catabolized or absorbed by *L. plantarum* (Table 2). Previous studies reported that *L. plantarum* has a complex enzyme system presenting the ability to degrade caffeine to theobromine and paraxanthine [8,34]. The slight decrease in caffeine in yeast mono-inoculation might be because it was degraded to theophylline and theobromine by cytochrome P450 [35].

Chlorogenic acid declined significantly in all samples (Table 2). The decline of chlorogenic acid might be because esterases hydrolyzed it into quinic acid and/or other phenolic acids (e.g., caffeic, ferulic, and *p*-coumaric acids). Co- and sequential inoculations showed significantly more reduction of chlorogenic acid compared with the respective mono-inoculation, indicating the synergistic effects of *L. thermotolerans* and *L. plantarum*.

As compared with unfermented samples, the content of caffeic acid significantly increased in yeast mono-inoculation, but significantly decreased in LAB mono-, co-, and sequential inoculation (Table 2). The increase in caffeic acid could be because of the release from chlorogenic acid by esterases from *L. thermotolerans* [9], while the decrease in the other three treatments could be attributed to the metabolism by *L. plantarum* to generate vinyl derivatives (e.g., 4-vinyl catechol) by PadA decarboxylase [36]. It is notable that the changes in ferulic acid and *p*-coumaric acid are similar. They might be released from chlorogenic acid hydrolysis by yeast, followed by decarboxylation of *p*-coumaric, caffeic, and ferulic acids by decarboxylases of *L. plantarum* to 4-vinylphenol, 4-vinylcatechol, and 4-vinylguaiacol, respectively, then reduction to 4-ethylphenol, 4-ethylcatechol, and 4-ethylguaiacol, correspondingly [36,37].

Alkaloids (caffeine and trigonelline) and phenolic acids are free radical scavengers [38]. Two in vitro antioxidant capacity assays were conducted to assess the antioxidant capacity changes before and after the fermentation of SCG hydrolysates. In general, the antioxidant capacity stayed relatively stable in terms of ORAC assay; for the DPPH assay, a significant reduction of the antioxidant capacity was detected in samples involving *L. plantarum* ML Prime, but not the yeast mono-inoculation (Table 2), which may be ascribed to the larger extent of phenolic acid degradation by LAB, as discussed above. The discrepancy between the two assays may be due to the fact that the ORAC assay is more efficient to detect a wider range of free radicals, which may include other antioxidants that were unquantified (Table 2) [39].

### 3.6. Changes in Amino Compounds

**Figure 4:**
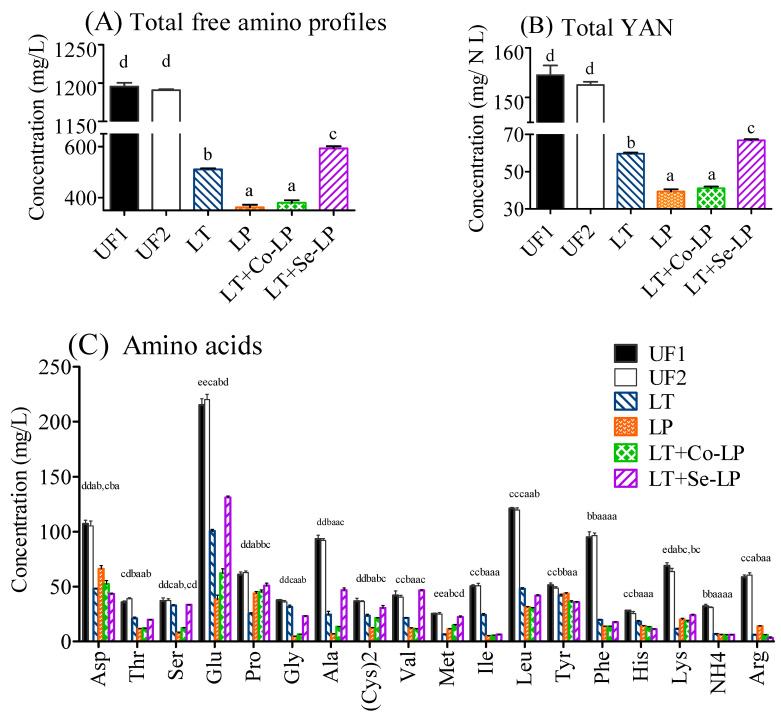
Total free amino profiles (**A**), total yeast assimilable nitrogen (YAN) (**B**), and specific amino compounds (**C**) in SCG hydrolysates at day 0 and day 14. UF1 and UF2: Unfermented SCG hydrolysates at D0 and D14; LT: *L. thermotolerans* Concerto; LP: *L. plantarum* ML Prime; LT+Co-LP: simultaneous inoculation of *L. thermotolerans* Concerto and *L. plantarum* ML Prime; LT+Se-LP: *L. thermotolerans* Concerto with sequentially inoculated *L. plantarum* ML Prime at day 4. a, b, c, d, e: Statistical analysis using ANOVA (*n* = 3) at 95% confidence interval was conducted in the same row, different letter indicated significant difference.

The total content of free amino acids was 1195.53 mg/L in the SCG hydrolysate medium before fermentation (Figure 4A, Table S1). The starting YAN in SCG hydrolysates was 154.51 N mg/L, which is close to the standard criteria as stated in grape wine fermentation (140.00 N mg/L) (Figure 4B) [40].

In general, all measured amino acids decreased after fermentation, especially phenylalanine, although different amino acids showed different reduction amounts (Figure 4C). The samples fermented with yeast (LT, LT+Co-LP, and LT+Se-LP) consumed more aspartic acid, proline, and lysine, while the samples fermented with *L. plantarum* ML Prime (LP, LT+Co-LP, and LT+Se-LP) metabolized more threonine, glycine, alanine, valine, leucine, isoleucine, and phenylalanine. The various amino acid assimilations by *L. thermotolerans* and *L. plantarum* ML Prime were due to their different needs for its respective biomass growth [4,9,28] for the formation of protein and other nitrogen-containing compounds, for the generation of certain volatile compounds such as high alcohols through the Ehrlich pathway, or for the production of some keto-acids [8,41].

### 3.7. Changes in Volatiles and PCA Analysis

#### 3.7.1. Changes in Volatile Components

**Figure 5:**
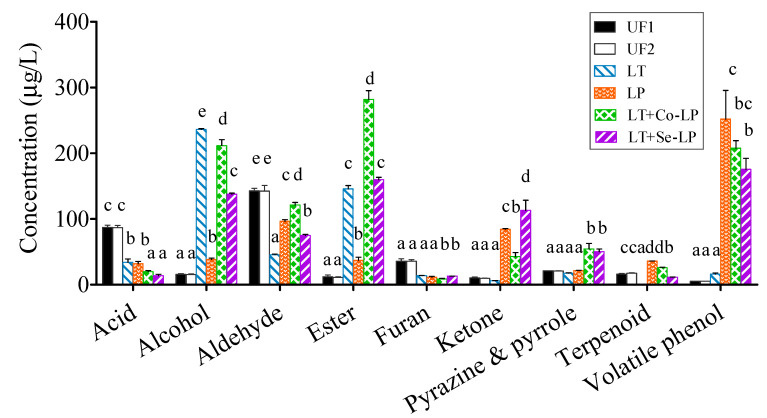
Subtotal contents of each type of volatiles. UF1 and UF2: Unfermented SCG hydrolysates at D0 and D14; LT: *L. thermotolerans* Concerto; LP: *L. plantarum* ML Prime; LT+Co-LP: simultaneous inoculation of *L. thermotolerans* Concerto and *L. plantarum* ML Prime; LT+Se-LP: *L. thermotolerans* Concerto with sequentially inoculated *L. plantarum* ML Prime at day 4. a, b, c, d, e: Statistical analysis using ANOVA (*n* = 3) at 95% confidence interval was conducted in the same row, different letter indicated significant difference.

A total of 72 volatile compounds were detected and quantified, including nine classes based on their functional groups, namely acids (twelve), alcohols (eight), aldehydes (eight), esters (seventeen), furans (five), ketones (five), pyrazines and pyrroles (six), terpenoids (four), and volatile phenols (seven), as shown in Table S1. In general, furans (e.g., 2,5-dimethylfuran and 2-acetylfuran) and aldehydes (e.g., 3-methyl pentanal and benzaldehyde) originated from SCG and were the dominant volatile compounds in unfermented SCG hydrolysates, while esters (e.g., ethyl acetate, ethyl decanoate, and isopropyl lactate) and alcohols (e.g., 2-phenylethyl alcohol and 2-ethyl-2-heptanol) were generated during fermentation and were the most abundant volatiles in fermented SCG hydrolysates (Figure 5, Table S2).

**Figure 6:**
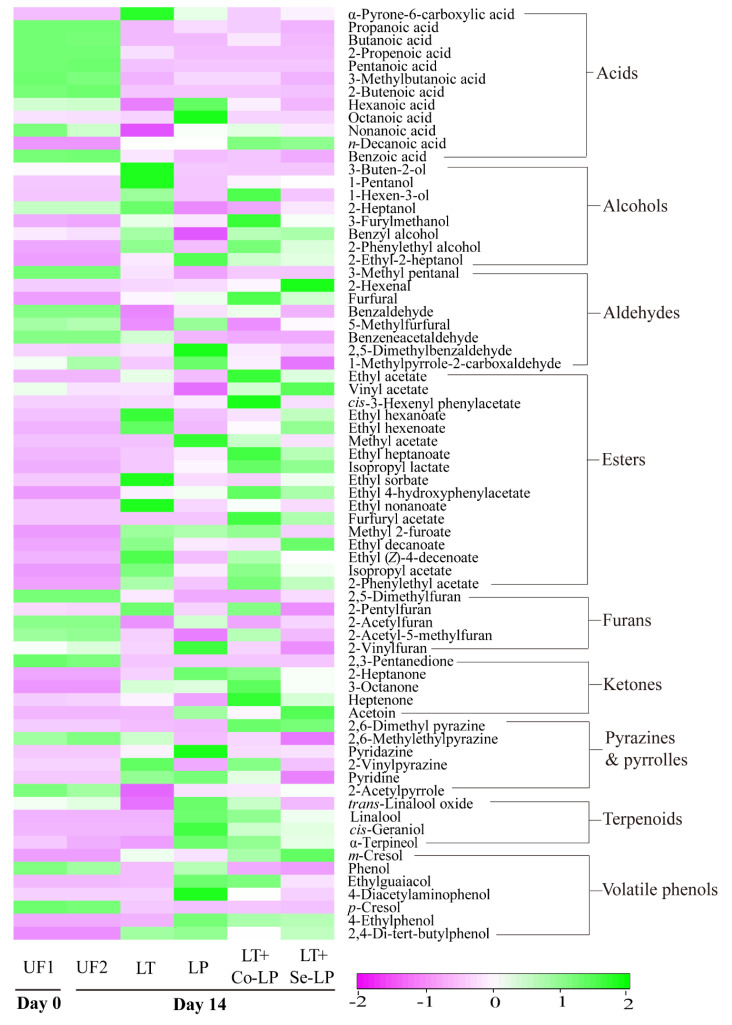
Heatmap of volatile profiles of SCG hydrolysates fermented with *L. thermotolerans* Concerto and *L. plantarum* ML Prime. UF1 and UF2: Unfermented SCG hydrolysates at D0 and D14; LT: *L. thermotolerans* Concerto; LP: *L. plantarum* ML Prime; LT+Co-LP: simultaneous inoculation of *L. thermotolerans* Concerto and *L. plantarum* ML Prime; LT+Se-LP: *L. thermotolerans* Concerto with sequentially inoculated *L. plantarum* ML Prime at day 4.

In general, most fatty acids declined to low or trace levels after fermentation (Figure 6 and Table S2). This may be expected, as fatty acids are the precursors to form corresponding esters. Interestingly, α-pyrone-6-carboxylic acid and n-decanoic acid were produced after fermentation, which agreed with our previous study [9], where the two fatty acids were also produced in SCG hydrolysates fermented with *L. thermotolerans* and *O. oeni* in the early stage of fermentation.

Nine alcohols (mostly products of the reduction of aldehydes) were detected in the fermented samples with varied amounts (Figure 6, Table S2), with higher levels in fermentations involving yeast. 2-Phenylethyl alcohol (a product of phenylalanine catabolism; floral, sweet, rosy) was generated in all samples with the highest amount in co-inoculation, followed by yeast mono-inoculation, sequential inoculation, and LAB mono-inoculation (Table S2), indicating the key role of yeast in its formation. It is interesting to note that 1-pentanol (balsamic, sweet) significantly increased in samples involving yeast inoculation (Figure 6, Table S2), indicating *L. thermotolerans* is a key producer rather than the bacteria. Samples with *L. plantarum* ML Prime, especially LAB mono-inoculation (LP), produced more 2-ethyl-2-heptanol, demonstrating that the bacteria contributed more to its production. In addition, 3-buten-2-ol and 1-hexen-3-ol presented a higher production in yeast mono-inoculation, both of which were likely formed from the reduction of their precursor aldehydes. 3-Furylmethanol (a reduced product of 5-methylfurfural) showed the highest content in co-inoculation and may contribute to coffee flavor.

Most aldehydes significantly decreased after fermentation (Figure 4B), which could be transformed to the corresponding alcohols and/or oxidized to the corresponding acids (Figure 6, Table S2). Interestingly, the amount of furfural remarkably raised in all samples, with significantly higher levels in mixed-inoculation (Figure 6, Table S2). 5-Methylfurfural remained relatively stable in LAB mono-inoculation, but significantly declined in samples involving yeast. Furfural could be released from its bound form in SCG hydrolysates after fermentation; this result was in line with the findings of SCG hydrolysates fermented with *L. thermotolerans* and *O. oeni* [9].

A total of 17 esters were identified in fermented samples, with vinyl acetate being the only ester detected in unfermented control (Figure 6, Table S2). The concentration of esters was the highest in co-inoculation samples, followed by sequential, yeast mono-, and LAB mono-inoculation (Figure 5). Ethyl acetate, *cis*-3-hexenyl phenylacetate, ethyl heptanoate, isopropyl lactate, and 2-phenylethyl acetate were in the highest content in co-inoculation (Figure 6), which might be because of the metabolic interactions of both the yeast and *L. plantarum* ML Prime [15]. Ethyl decanoate, with floral and fruity notes, was mainly produced by *L. thermotolerans*, with higher contents in yeast mono- and sequential inoculation (Figure 6). It is also interesting to note that isopropyl lactate was produced after fermentation with a higher amount in mixed inoculation involving LAB (Table S2). Furfuryl acetate and methyl 2-furoate were two esters formed from respective precursors, furfuryl alcohol and 2-furancarboxylic acid, in SCG hydrolysates.

The abundant furans such as 2,5-dimethyfuran and 2-acetyfuran decreased to low or undetectable levels after fermentation (Figure 6, Table S2). 2-Vinylfuran remained relatively stable in all treatments. For 2-pentylfuran, only a significant increase was observed in the yeast mono-inoculation, while it remained stable in other samples. Differently, 2-acetyl-5-methylfuran remained stable in co-inoculation, but showed an obvious decrease in other samples.

Among the five ketones, four significantly increased and one ketone (2,3-pentanedionde) decreased to undetectable levels after fermentation (Figure 6, Table S2). Acetoin was quantitatively the predominant ketone and had the highest amounts in inoculations involving *L. plantarum*; this is associated with its citrate catabolism.

Pyrazines and pyrroles with coffee origin are key volatiles for coffee aroma. Pyrazines presented different changing trends (Figure 6, Table S2). For example, 2,6-dimethyl pyrazine significantly increased in co- and sequential inoculation, but slightly decreased in the two mono-inoculations.

Four terpenoids were quantified in all samples (Figure 6, Table S2). The increase in *cis*-geraniol, linalool, and α-terpineol was only observed in samples involving *L. plantarum* ML Prime. While *trans*-linalool oxide significantly decreased in yeast mono- and sequential inoculation, it significantly increased in LAB mono- and sequential inoculation (Figure 6, Table S2). The increases in these oxygenated terpenes were likely due to their release from the bound glycosides upon hydrolysis by LAB glycosidases.

Four volatile phenols were identified in the unfermented SCG hydrolysates and three newly produced volatile phenols were also detected after fermentation (Figure 6, Table S2). Among the existent volatile phenols, 4-ethyl guaiacol increased after fermentation with significantly higher amounts in samples containing *L. plantarum* ML Prime. 4-Ethyl guaiacol could be produced by *L. plantarum* via decarboxylating ferulic acid as discussed above. Additionally, a large amount of 4-ethy phenol was produced in LAB-inoculated samples as a result of the decarboxylation of *p*-coumaric acid by this LAB mentioned above. The production of volatile phenols would contribute to the perception of the smoky flavor of coffee.

#### 3.7.2. PCA

**Figure 7:**
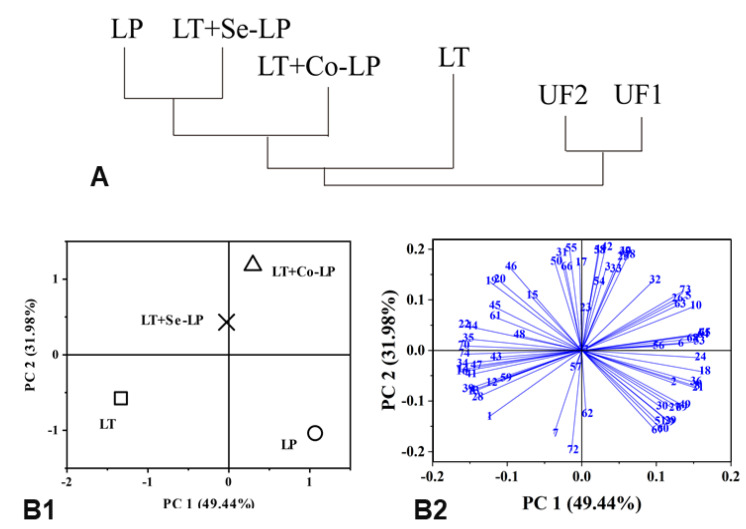
Cluster relationship (**A**) and PCA analysis of volatile profiles (**B**) of SCG hydrolysates fermented with *L. thermotolerans* Concerto and *L. plantarum* ML Prime. UF1 and UF2: Unfermented SCG hydrolysates at D0 and D14; LT (□): *L. thermotolerans* Concerto; LP (○): *L. plantarum* ML Prime; LT+Co-LP (**△**): simultaneous inoculation of *L. thermotolerans* Concerto and *L. plantarum* ML Prime; LT+Se-LP (X): *L. thermotolerans* Concerto with sequentially inoculated *L. plantarum* ML Prime at day 4. The compound names (1-72) were listed in Table S3.

PCA (focusing on fermented samples) was conducted using the selected volatile profiles in Table 1 and Table S2 to ascertain the relationship between the treatments and the production of volatile compounds (Figure 7). The first two principal components (PC) accounted for 81.42% of the total variance. PC1 comprised 49.44% and distinguished yeast mono-inoculation (LT) and sequential inoculation from *L. plantarum* mono- and co-inoculation (Figure 7B1). The yeast mono- and sequential inoculation on the same left negative side of PC1 might be due to their higher content of ethanol and some endogenous compounds such as furans (#47, 2,5-dimethylfuran; #50, 2-acetyl-5-methylfuran, Figure 7B2, Tables S2 and S3) and pyrazines (#59, 2,6-methylethylpyrazine; #61, 2-vinylpyrazine, Figure 7B2, Tables S2 and S3). PC2 comprised 31.98% and distinguished mono-inoculation from mixed inoculation, which is also indicated by the clustering of all treatments (Figure 7A). This might be because mixed inoculation had higher levels of higher alcohols (#20, 2-phenylethyl alcohol, Figure 7B2, Tables S2 and S3), aldehydes (#22, 3-methyl pentanal; #23, 2-hexenal, Figure 7B2, Tables S2 and S3) and esters (#31, ethyl acetate; #35, ethyl hexenoate; #39, ethyl sorbate; #41, ethyl nonanoate; #45, isopropyl acetate, Figure 7B2, Tables S2 and S3). The PCA plots demonstrated that co- and sequential inoculation transformed the volatile profiles in contrast with mono-inoculation. Therefore, mixed inoculation could be an alternative way to valorize SCG via fermentation.

## 4. Conclusions

In conclusion, mono-, co-, and sequential inoculations of *L. plantarum* ML Prime and *L. thermotolerans* were investigated in SCG hydrolysates fermentation. Our results showed that *L. plantarum* inhibited the growth and performance of *L. thermotolerans* in mixed inoculations. In addition, less succinic, lactic, and acetic acids were found in sequential inoculation as compared with co-inoculation. Moreover, *L. plantarum* showed a strong ability to catabolize phenolic acids, especially caffeic, ferulic, and *p*-coumaric acids, leading to lower phenolic acid contents in co-inoculation than those in sequential inoculation. Furthermore, the amount of esters was higher in co-inoculation than that in sequential inoculation. The complex interactions between *L. thermotolerans* and *L. plantarum* on different volatile changes require further investigation.

## Figures and Tables

**Table 1:** Oenological parameters of SCG hydrolysates fermented with *L. thermotolerans* and *L. plantarum*.

	Day 0	Day 14				
	UF1	UF2	LT	LP	LT+Co-LP	LT+Se-LP
pH	5.00 ± 0.00 d	5.00 ± 0.00 d	4.93 ± 0.02 c	3.94 ± 0.01 a	3.96 ± 0.01 a	4.13 ±0.01 c
°Brix	15.02 ± 0.01 e	15.00 ± 0.01 e	8.90 ± 0.02 a	14.17 ± 0.04 d	12.70 ± 0.05 c	9.69 ± 0.03 b
Ethanol (%, *v/v*)	0.00 ± 0.00 a	0.00 ± 0.00 a	4.92 ± 0.28 c	0.00 ± 0.00 a	1.03 ± 0.01 b	3.20 ± 0.14 c
Sugars (g/L)						
Fructose	12.65 ± 0.48 b	12.36 ± 0.06 b	3.53 ± 0.04 a	3.58 ± 0.15 a	3.47 ± 0.17 a	3.33 ± 0.29 a
Glucose	47.33 ± 1.67 d	47.31 ± 0.47 d	4.40 ± 0.12 a	12.02 ± 0.82 c	7.40 ± 0.08 b	3.53 ± 0.20 a
Sucrose	18.18 ± 1.06 c	18.29 ± 0.28 c	2.23 ± 0.03 a	3.94 ± 0.16 b	3.13 ± 0.10 ab	2.72± 0.02 ab
Mannose	20.17 ± 1.25 d	20.18 ± 0.22 d	5.31 ± 0.27 bc	3.86 ± 0.05 ab	5.93 ± 0.14 c	3.82 ± 0.05 a
Galactose	16.35 ± 0.31 e	16.37 ± 0.37 e	8.33 ± 0.17 d	7.37 ± 0.16 c	5.55 ± 0.09 b	4.50 ± 0.22 a
Arabinose	2.46 ± 0.06 b	2.49 ± 0.08 b	2.41 ± 0.09 b	2.38 ± 0.08 b	2.39 ± 0.05 b	2.05 ± 0.03 a
Total	117.25 ± 1.01 e	117.11 ± 0.56 e	26.26 ± 0.32 b	33.21 ± 0.70 d	27.94 ± 0.24 c	20.37 ± 0.08 a
Glycerol (g/L)	0.00 ± 0.00 a	0.00 ± 0.00 a	4.27 ± 0.25 d	0.00 ± 0.00 a	1.07 ± 0.04 b	2.52 ± 0.17 c
Organic acids (g/L)						
Citric acid	38.16 ± 1.33 c	38.26 ± 1.25 c	34.39 ± 0.81 b	28.91 ± 0.06 a	29.24 ± 0.32 a	27.02 ± 0.87 a
α-Ketoglutaric acid (mg/L)	33.70 ± 1.27 b	31.37 ± 2.98 b	19.72 ± 0.70 a	18.04 ± 1.16 a	31.79 ± 0.97 a	34.36 ± 0.83 a
Malic acid	0.46 ± 0.04 b	0.45 ± 0.04 b	0.10 ± 0.00 a	0.08 ± 0.01 a	0.08 ± 0.01 a	0.08 ± 0.01 a
Pyruvic acid	0.09 ± 0.01 b	0.09 ± 0.00 b	0.26 ± 0.01 e	0.06 ± 0.00 a	0.14 ± 0.01 c	0.24 ± 0.00 d
Succinic acid	1.83 ± 0.04 a	1.85 ± 0.01 a	2.80 ± 0.05 b	4.68 ± 0.00 c	4.48 ± 0.08 c	2.96 ± 0.01 b
Lactic acid	0.14 ± 0.00 a	0.14 ± 0.01 a	0.58 ± 0.01 a	39.84 ± 1.42 c	42.92 ± 0.36 d	33.41 ± 0.60 b
Acetic acid	0.13 ± 0.00 a	0.13 ± 0.05 a	0.49 ± 0.02 b	3.96 ± 0.16 c	5.26 ± 0.19 d	3.81 ± 0.08 c
Total	40.37 ± 1.38 a	40.49 ± 1.26 a	38.55 ± 0.90 a	77.49 ± 1.64 c	82.07 ± 1.00 d	67.49 ± 1.56 b

Notes: UF1 and UF2: Unfermented SCG hydrolysates at day 0 and day 14; LT: mono-inoculation of *L. thermotolerans* Concerto; LP: mono-inoculation of *L. plantarum* ML Prime; LT+Co-LP: co-inoculation of *L. thermotolerans* Concerto and *L. plantarum* ML Prime; LT+Se-LP: sequential inoculation of *L. thermotolerans* Concerto and *L. plantarum* ML Prime at day 4. a, b, c, d, e: Statistical analysis using ANOVA (*n* = 3) at 95% confidence interval was conducted in the same row, different letter indicated significant difference.

**Table 2:** Changes in alkaloids, phenolic compounds, and antioxidant capacity in SCG hydrolysates before and after fermentation with *L. thermotolerans* and *L. plantarum*.

	Day 0	Day 14				
	UF1	UF2	LT	LP	LT+Co-LP	LT+Se-LP
Alkaloids						
Trigonelline	530.82 ± 4.07 b	529.69 ± 3.66 b	532.13 ± 5.48 b	518.24 ± 10.18 b	452.72 ± 5.72 a	507.62 ± 2.48 b
Caffeine	1181.26 ± 11.65 b	1178.82 ± 1.90 b	1151.97 ± 1.39 b	806.02 ± 7.69 a	800.26± 1.20 a	788.15 ± 2.46 a
Theobromine	124.75 ± 1.38 d	122.92 ± 2.10 d	40.92 ± 0.90 a	103.24 ± 1.32 c	100.15 ± 0.23 c	77.90 ± 1.05 b
Theophylline	73.66 ± 1.83 b	73.43 ± 1.51 b	33.97 ± 0.84 a	64.05 ± 0.07 b	64.92 ± 0.37 b	92.10 ± 3.75 c
Phenolic acids						
Chlorogenic acid	135.70 ± 1.58 d	135.13 ± 1.30 d	53.67 ± 1.29 b	120.33 ± 5.33 c	26.19 ± 0.45 a	24.69 ± 1.11 a
Caffeic acid	595.47 ± 5.67 b	596.58 ± 6.26 b	646.46 ± 13.56 c	16.20 ± 1.01 a	37.81 ± 1.40 a	11.31 ± 1.63 a
Ferulic acid	13.19 ± 0.20 b	13.06 ± 0.22 b	27.97 ± 1.78 c	7.66 ± 0.19 a	10.36 ± 0.28 ab	10.21 ± 0.70 ab
*p*-Coumaric acid	7.55 ± 0.02 c	7.60 ± 0.05 c	11.85 ± 0.11 d	6.04 ± 0.02 b	5.65 ± 0.06 a	6.16 ± 0.01 b
Antioxidant capacity						
DPPH (μmol TE/L)	4.58 ± 0.10 c	4.31 ± 0.13 c	4.74 ± 0.37 c	3.61 ± 0.27 b	3.34 ± 0.01 a	3.66 ± 0.22 b
ORAC (mmol TE/L)	707.00 ± 42.22 a	687.61 ± 88.99 a	756.56 ± 92.71 a	827.05 ± 74.28 a	703.67 ± 1.66 a	684.27 ± 83.52 a

Notes: UF1 and UF2: Unfermented SCG hydrolysates at day 0 and day 14; LT: mono-inoculation of *L. thermotolerans* Concerto; LP: mono-inoculation of *L. plantarum* ML Prime; LT+Co-LP: co-inoculation of *L. thermotolerans* Concerto and *L. plantarum* ML Prime; LT+Se-LP: sequential inoculation of *L. thermotolerans* Concerto and *L. plantarum* ML Prime at day 4. a, b, c, d: Statistical analysis using ANOVA (*n* = 3) at 95% confidence interval was conducted in the same row, different letter indicated significant difference. TE: Trolox equivalent.

## Data Availability

Data is contained within the article or supplementary material.

## Supplementary Materials

The following supporting information can be downloaded at: https://www.mdpi.com/article/10.3390/foods12061161/s1, Table S1: Amino acids and ammonia in unfermented and fermented SCG hydrolysates; Table S2: Changes in volatile compounds of SCG hydrolysates before and after fermentation; Table S3: Compound names related to numbers used in PCA analysis.
