# Supplementary material for: Transforming Spent Coffee Grounds’ Hydrolysates with Yeast Lachancea thermotolerans and Lactic Acid Bacterium Lactiplantibacillus plantarum to Develop Potential Novel Alcoholic Beverages
Source: Foods. 2023 Mar 9;12(6):1161. doi: 10.3390/foods12061161 (PMC10048607; doi:10.3390/foods12061161)
Supplement: Supplementary file 1 [file foods-12-01161-s001.zip › foods-2239513-supplementary.pdf]

Supporting information

Transforming spent coffee grounds hydrolysates with yeast *Lachancea thermotolerans* and lactic acid bacterium *Lactiplantibacillus plantarum* to develop potential novel alcoholic beverages

Yunjiao Liu [a], Yuyun Lu [a*], Shao Quan Liu [a,b,*]

[a] Department of Food Science and Technology, Science Drive 2, Faculty of Science, National University of Singapore, Singapore 117542, Singapore.

[b] National University of Singapore (Suzhou) Research Institute, 377 Lin Quan Street, Suzhou Industrial Park, Jiangsu 215123, China.

Corresponding Author

* Department of Food Science and Technology, Science Drive 2, National University of Singapore, Singapore 117543, Singapore. Telephone: 65-6516 2687. Fax: 65-6775-7895. E-mail: fstluy@nus.edu.sg (Yuyun Lu); fstlsq@nus.edu.sg (Shao Quan Liu)

Table S1. Amino acids and ammonia in unfermented and fermented SCG hydrolysates.

Name (mg/L)	Short Name	Day 0	Day 14				
		UF1	UF2	LT	LP	LT+Co-LP	LT+Se-LP
Aspartic acid	Asp	107.10 ± 3.27d	105.35 ± 4.40d	48.09 ± 0.09ab	66.12 ± 3.10c	52.43 ± 3.10b	43.28 ± 0.71a
Threonine	Thr	35.69 ± 1.16c	39.08 ± 0.84d	20.93 ± 1.33b	11.62 ± 0.07a	12.00 ± 0.07a	19.78 ± 0.15b
Serine	Ser	36.95 ± 2.69d	37.33 ± 1.55d	32.89 ± 0.76c	7.71 ± 0.81a	12.05 ± 0.81b	33.49 ± 0.10cd
Glutamic acid	Glu	215.06 ± 5.96e	220.25 ± 4.68e	100.62 ± 1.44c	38.34 ± 3.90a	62.29 ± 3.90b	131.27 ± 1.35d
Proline	Pro	60.90 ± 2.36d	62.82 ± 1.37d	25.17 ± 0.97a	43.81 ± 1.78b	45.17 ± 1.78b	50.90 ± 2.28c
Glycine	Gly	37.52 ± 0.75d	36.02 ± 1.30d	31.41 ± 1.71c	4.60 ± 0.12a	6.44 ± 0.12a	22.91 ± 0.58b
Alanine	Ala	93.27 ± 3.59d	92.07 ± 1.64d	24.45 ± 3.13b	6.91 ± 0.18a	13.48 ± 0.18a	46.83 ± 1.85c
Cystine	(Cys)2	36.55 ± 2.74d	36.46 ± 0.83d	23.48 ± 1.32b	12.42 ± 0.25a	21.42 ± 0.25b	30.50 ± 2.30c
Valine	Val	41.85 ± 4.13c	40.37 ± 1.73c	21.42 ± 0.53b	11.84 ± 0.60a	11.13 ± 0.60a	46.41 ± 0.84c
Methionine	Met	25.19 ± 0.63e	24.92 ± 1.45e	6.50 ± 0.46a	11.18 ± 0.54b	14.79 ± 0.54c	22.14 ± 1.13d
Isoleucine	Ile	50.24 ± 1.49c	50.64 ± 2.31c	24.08 ± 1.41b	5.06 ± 0.52a	5.34 ± 0.52a	6.31 ± 0.41a
Leucine	Leu	121.05 ± 1.12c	119.85 ± 1.75c	47.94 ± 1.13c	31.24 ± 0.71a	30.25 ± 0.71a	41.83 ± 0.65b
Tyrosine	Tyr	51.36 ± 1.87c	48.62 ± 1.37c	41.81 ± 1.37b	43.59 ± 0.98b	36.07 ± 0.98a	35.75 ± 0.76a
Phenylalanine	Phe	95.05 ± 4.99b	96.47 ± 2.50b	19.61 ± 0.59a	13.74 ± 0.15a	13.74 ± 0.15a	17.52 ± 0.43a
Histidine	His	28.12 ± 0.48c	25.50 ± 1.94c	18.17 ± 0.96b	13.97 ± 0.52a	13.08 ± 0.52a	11.16 ± 0.44a
Lysine	Lys	68.95 ± 2.84e	63.74 ± 2.87d	11.32 ± 0.68a	19.90 ± 1.19bc	18.16 ± 1.19b	23.88 ± 0.87c
ammonium	NH4	32.02 ± 1.92b	30.83 ± 0.58b	6.69 ± 0.39a	6.15 ± 0.42a	6.01± 0.42a	6.13 ± 0.18a
Arginine	Arg	58.65 ± 1.88c	60.39 ± 2.21c	6.16 ± 0.33a	13.90 ± 0.44b	5.86 ± 0.44a	3.16 ± 0.96a
∑ AA		1195.53 ± 4.92d	1190.73 ± 1.25d	510.76 ± 4.25b	362.11 ± 10.39a	379.80 ± 10.13a	593.24 ± 8.34c
∑YAN (N mg/L)		154.51± 1.93d	152.53 ± 0.64d	59.66 ± 0.68b	39.35 ± 1.23a	41.08 ± 0.90a	66.88 ± 0.57c

Notes: UF1 and UF2: Unfermented SCG hydorlysates at day 0 and day 14; LT: *L. thermotolerans* Concerto, LP: *L. plantarum* ML Prime, LT+Co-LP: simultaneous inoculation of *L. thermotolerans* Concerto and *L. plantarum* ML Prime; LT+Se-LP: *L. thermotolerans* Concerto with sequentially inoculated *L. plantarum* ML Prime at day 4. a, b, c, d, e: Statistical analysis using ANOVA (n=3) at 95% confidence interval. Same letters indicate no significant difference between samples.

Table S2. Changes in volatile compounds of SCG hydrolysates before and after fermentation.

	Identification		Day 0	Day 14				
**Compound (μg/L)**	**methods**	**LRI**	**UF1**	**UF2**	**LT**	**LP**	**LT+Co-LP**	**LT+Se-LP**
**Acids**								
α-Pyrone-6-carboxylic acid	MS, LRI	1365	0.00 ± 0.00a	0.00 ± 0.00a	26.06 ± 4.5e	9.54 ± 0.1d	1.65 ± 0.01b	5.66 ± 1.27c
Propanoic acid	MS, LRI	1534	7.77 ± 1.75c	7.72 ± 0.26c	0.46 ± 0.06a	1.79 ± 0.25b	1.17 ± 0.29b	0.11 ± 0.01a
Butanoic acid	MS, LRI	1623	8.3 ± 2.11c	8.25 ± 0.40c	0.00 ± 0.00a	0.00 ± 0.00a	2.11 ± 0.4b	0.00 ± 0.00a
2-Propenoic acid	MS, LRI	1635	0.98 ± 0.14c	0.99 ± 0.08c	0.18 ± 0.00b	0.00 ± 0.00a	0.00 ± 0.00a	0.00 ± 0.00a
Pentanoic acid	MS, LRI	1647	5.14 ± 0.05b	5.24 ± 0.18b	0.86 ± 0.14a	0.95 ± 0.07a	0.98 ± 0.10a	0.93 ± 0.12a
3-Methylbutanoic acid	MS, LRI	1664	26.08 ± 3.03c	24.85 ± 2.76c	1.98 ± 0.32ab	6.78 ± 1.00b	6.65 ± 0.76ab	1.91 ± 0.24a
2-Butenoic acid	MS, LRI	1774	29.03 ± 1.22c	30.01 ± 0.58c	0.46 ± 0.08b	0.42 ± 0.08b	0.24 ± 0.04a	0.15 ± 0.03a
Hexanoic acid	MS, LRI	1840	5.73 ± 1.02cd	5.76 ± 1.08cd	2.03 ± 0.4a	7.78 ± 0.97d	4.48 ± 0.8bc	3.25 ± 0.31ab
Octanoic acid	MS, LRI	2033	0.20 ± 0.01b	0.17 ± 0.02b	0.00 ± 0.00a	3.30 ± 0.24c	0.00 ± 0.00a	0.00 ± 0.00a
Nonanoic acid	MS, LRI	2144	0.57 ± 0.02b	0.50 ± 0.05b	0.28 ± 0.05a	0.46 ± 0.08b	0.48 ± 0.07b	0.43 ± 0.05b
n-Decanoic acid	MS, LRI	2266	0.00 ± 0.00a	0.00 ± 0.00a	0.67 ± 0.07b	0.65 ± 0.11b	1.32 ± 0.22c	1.25 ± 0.25c
Benzoic acid	MS, LRI	2442	3.34 ± 0.20c	3.37 ± 0.34c	1.20 ± 0.38b	0.51 ± 0.63a	0.62 ± 0.11a	0.26 ± 0.22a
**Alcohols**								
3-Buten-2-ol	MS, LRI	1125	1.78 ± 0.28a	1.8 ± 0.27a	4.06 ± 0.52b	1.22 ± 0.06a	1.18 ± 0.06a	1.18 ± 0.2a
1-Pentanol	MS, LRI	1202	1.42 ± 0.16b	1.39 ± 0.15b	53.24 ± 6.03d	0.00 ± 0.00a	10.5 ± 2.12c	12.35 ± 1.53c
1-Hexen-3-ol	MS, LRI	1247	0.00 ± 0.00a	0.00 ± 0.00a	0.20 ± 0.01b	0.00 ± 0.00a	0.29 ± 0.09b	0.00 ± 0.00a
2-Heptanol	MS, LRI	1316	9.27 ± 0.51bc	9.32 ± 0.61bc	10.7 ± 1.80c	6.18 ± 0.02a	6.72 ± 0.01a	7.86 ± 1.05ab
3-Furylmethanol	MS, LRI	1671	0.87 ± 0.00a	0.62 ± 0.13a	3.84 ± 0.53b	2.55 ± 0.32b	8.01 ± 1.23c	3.4 ± 0.12b
Benzyl alcohol	MS, LRI	1897	2.44 ± 0.25b	2.30 ± 0.17b	3.57 ± 0.06c	0.82 ± 0.10a	3.40 ± 0.08c	3.52 ± 0.15c
2-Phenylethyl alcohol	MS, LRI	1940	0.00 ± 0.00a	0.00 ± 0.00a	158.6 ± 5.04d	18.29 ± 0.66b	175.9 ± 6.60e	104.81 ± 1.30c
2-Ethyl-2-heptanol	MS, LRI	2135	0.00 ± 0.00a	0.00 ± 0.00a	2.05 ± 0.38b	6.67 ± 0.81d	3.87 ± 0.75c	3.27 ± 0.48bc
**Aldehydes**								
3-Methyl pentanal	MS, LRI	1080	5.82 ± 0.77b	5.82 ± 0.77b	3.14 ± 0.54a	1.95 ± 0.19a	2.62 ± 0.31a	2.52 ± 0.20a
2-Hexenal	MS, LRI	1429	0.00 ± 0.00a	0.00 ± 0.00a	0.13 ± 0.01b	0.17 ± 0.01b	0.51 ± 0.08c	2.97 ± 0.46d
Furfural	MS, LRI	1473	1.44 ± 0.1a	1.34 ± 0.05a	12.58 ± 0.38b	15.32 ± 0.77c	33.22 ± 1.05e	19.21 ± 2.02d
Benzaldehyde	MS, LRI	1533	118.28 ± 3.65e	118.02 ± 3.48e	23.87 ± 0.31a	62.01 ± 0.08c	82.22 ± 0.44c	41.57 ± 0.11b
5-Methylfurfural	MS, LRI	1593	10.76 ± 1.52c	10.03 ± 1.05c	0.42 ± 0.02a	11.21 ± 1.06c	0.58 ± 0.01a	6.13 ± 0.20b
Benzeneacetaldehyde	MS, LRI	1654	4.85 ± 0.29d	4.84 ± 0.40d	3.31 ± 0.72c	0.46 ± 0.00b	0.20 ± 0.00a	0.21 ± 0.05a
2,5-Dimethylbenzaldehyde	MS, LRI	1830	1.27 ± 0.17a	1.21 ± 0.21a	1.54 ± 0.21a	4.89 ± 0.14b	1.63 ± 0.25a	1.28 ± 0.18a
1-Methylpyrrole-2-carboxaldehyde	MS, LRI	2119	0.40 ± 0.07a	0.45 ± 0.11a	0.35 ± 0.04a	0.49 ± 0.11a	0.38 ± 0.05a	0.29 ± 0.04a
**Esters**								
Ethyl acetate	MS, LRI	/	0.00 ± 0.00a	0.00 ± 0.00a	88.6 ± 0.67c	5.65 ± 0.40b	227.59 ± 11.43e	98.47 ± 0.05d
Vinyl acetate	MS, LRI	1021	12.18 ± 2.44abc	11.04 ± 1.1bc	11.11 ± 0.18a	8.32 ± 1.34b	12.75 ± 1.71cd	15.21 ± 0.49d
cis-3-Hexenyl phenylacetate	MS, LRI	1158	0.00 ± 0.00a	0.00 ± 0.00a	0.09 ± 0.00a	0.38 ± 0.05b	3.77 ± 0.53c	0.22 ± 0.00b
Ethyl hexanoate	MS, LRI	1216	0.00 ± 0.00a	0.00 ± 0.00a	5.04 ± 0.45d	0.00 ± 0.00a	0.74 ± 0.1b	2.64 ± 0.53c
Ethyl hexenoate	MS, LRI	1296	0.00 ± 0.00a	0.00 ± 0.00a	5.21 ± 0.60e	0.14 ± 0.01b	1.78 ± 0.16c	4.18 ± 0.45d
Methyl acetate	MS, LRI	1309	0.00 ± 0.00a	0.00 ± 0.00a	0.00 ± 0.00a	10.69 ± 2.18d	5.14 ± 0.98c	1.66 ± 0.05b
Ethyl heptanoate	MS, LRI	1319	0.00 ± 0.00a	0.00 ± 0.00a	0.39 ± 0.08b	1.05 ± 0.00c	4.55 ± 0.58e	2.65 ± 0.33d
Isopropyl lactate	MS, LRI	1324	0.00 ± 0.00a	0.00 ± 0.00a	0.39 ± 0.08b	1.05 ± 0.00c	3.13 ± 0.16e	2.65 ± 0.33d
Ethyl sorbate	MS, LRI	1506	0.00 ± 0.00a	0.00 ± 0.00a	4.07 ± 0.62d	0.30 ± 0.02b	0.09 ± 0.00b	1.14 ± 0.14c
Ethyl 4-hydroxyphenylacetate	MS, LRI	1515	0.00 ± 0.00a	0.00 ± 0.00a	0.92 ± 0.04b	1.19 ± 0.00b	2.56 ± 0.18d	1.89 ± 0.16c
Ethyl nonanoate	MS, LRI	1525	0.00 ± 0.00a	0.00 ± 0.00a	5.14 ± 0.00d	0.35 ± 0.07b	1.31 ± 0.27c	0.48 ± 0.02b
Furfuryl acetate	MS, LRI	1537	0.00 ± 0.00a	0.00 ± 0.00a	0.00 ± 0.00a	0.00 ± 0.00a	0.44 ± 0.00c	0.26 ± 0.03b
Methyl 2-furoate	MS, LRI	1583	0.00 ± 0.00a	0.00 ± 0.00a	2.69 ± 0.45c	2.45 ± 0.45c	2.78 ± 0.48c	0.76 ± 0.04b
Ethyl decanoate	MS, LRI	1631	0.00 ± 0.00a	0.00 ± 0.00a	18.77 ± 2.11c	6.5 ± 0.77b	5.72 ± 0.82b	21.15 ± 2.19c
Ethyl (*Z*)-4-decenoate	MS, LRI	1657	0.00 ± 0.00a	0.00 ± 0.00a	11.98 ± 1.61e	0.55 ± 0.05b	7.68 ± 0.93d	3.95 ± 0.65c
Isopropyl acetate	MS, LRI	1787	0.00 ± 0.00a	0.00 ± 0.00a	1.41 ± 0.21c	0.54 ± 0.04b	1.35 ± 0.32c	0.75 ± 0.11b
2-Phenylethyl acetate	MS, LRI	1821	0.00 ± 0.00a	0.00 ± 0.00a	2.66 ± 0.10c	0.58 ± 0.11b	3.28 ± 0.37d	2.35 ± 0.48c
**Furans**								
2,5-Dimethylfuran	MS, LRI	1014	20.51 ± 2.95c	20.37 ± 0.86c	6.6 ± 0.52b	0.00 ± 0.00a	0.00 ± 0.00a	5.24 ± 0.44b
2-Pentylfuran	MS, LRI	1210	1.46 ± 0.04ab	1.44 ± 0.10ab	2.29 ± 0.42c	1.41 ± 0.30ab	2.19 ± 0.43bc	1.05 ± 0.15a
2-Acetylfuran	MS, LRI	1512	10.84 ± 1.07c	10.94 ± 1.49c	3.10 ± 0.51a	8.60 ± 0.95c	3.81 ± 0.73a	5.42 ± 0.04b
2-Acetyl-5-methylfuran	MS, LRI	1624	2.21 ± 0.19c	2.28 ± 0.09c	1.13 ± 0.14b	0.48 ± 0.06a	2.02 ± 0.15c	0.92 ± 0.09b
2-Vinylfuran	MS, LRI	1869	0.72 ± 0.14ab	0.77 ± 0.18ab	0.63 ± 0.11b	1.02 ± 0.14a	0.63 ± 0.11b	0.50 ± 0.02b
**Ketones**								
2,3-Pentanedione	MS, LRI	1056	9.52 ± 1.07b	8.77 ± 0.19b	0.00 ± 0.00a	0.00 ± 0.00a	0.00 ± 0.00a	0.00 ± 0.00a
2-Heptanone	MS, LRI	1193	0.55 ± 0.02a	0.55 ± 0.01a	1.57 ± 0.06b	5.74 ± 0.78d	5.16 ± 0.33d	2.88 ± 0.33c
3-Octanone	MS, LRI	1247	0.00 ± 0.00a	0.00 ± 0.00a	0.18 ± 0.03b	0.17 ± 0.03b	0.31 ± 0.03c	0.14 ± 0.00b
Heptenone	MS, LRI	1238	0.57 ± 0.11ab	0.59 ± 0.02ab	0.93 ± 0.06bc	0.19 ± 0.01a	2.62 ± 0.58d	1.38 ± 0.20c
Acetoin	MS, LRI	1292	0.00 ± 0.00a	0.00 ± 0.00a	0.72 ± 0.06b	75.68 ± 1.63d	32.02 ± 4.74c	108.05 ± 14.99e
**Pyrazines & pyrroles**								
2,6-Dimethyl pyrazine	MS, LRI	1351	5.48 ± 0.73c	5.61 ± 0.82c	3.01 ± 0.42b	1.60 ± 0.12a	39.36 ± 7.89d	37.11 ± 3.2d
2,6-Methylethylpyrazine	MS, LRI	1385	0.79 ± 0.08c	0.86 ± 0.07c	0.69 ± 0.03c	0.41 ± 0.13ab	0.48 ± 0.01b	0.24 ± 0.03a

Pyridazine	MS, LRI	1440	0.00 ± 0.00a	0.00 ± 0.00a	0.93 ± 0.61c	5.39 ± 0.00d	0.41 ± 0.01b	0.54 ± 0.02b
2-Vinylpyrazine	MS, LRI	1443	0.61 ± 0.00a	0.61 ± 0.06a	1.25 ± 0.07b	0.46 ± 0.06a	1.14 ± 0.13b	0.55 ± 0.01a
Pyridine	MS, LRI	1501	1.57 ± 0.25b	1.58 ± 0.22b	3.10 ± 0.00d	3.34 ± 0.08d	2.4 ± 0.25c	0.87 ± 0.13a
2-Acetylpyrrole	MS, LRI	1982	12.71 ± 0.55cd	12.22 ± 1.00cd	8.57 ± 0.25a	10.49 ± 0.55b	10.56 ± 0.29bc	11.05 ± 0.66bc
**Terpenoids**								
trans-Linalool oxide	MS, LRI	1438	15.85 ± 2.79c	17.44 ± 4.81c	0.48 ± 0.09a	27.82 ± 0.89d	19.72 ± 2.61c	7.56 ± 0.5b
Linalool	MS, LRI	1539	0.00 ± 0.00a	0.00 ± 0.00a	0.00 ± 0.00a	4.96 ± 0.43c	4.36 ± 0.74c	2.28 ± 0.31b
cis-Geraniol	MS, LRI	1857	0.00 ± 0.00a	0.00 ± 0.00a	0.00 ± 0.00a	2.80 ± 0.54c	1.43 ± 0.14b	1.18 ± 0.23b
α-Terpineol	MS, LRI	1661	0.23 ± 0.04a	0.21 ± 0.02a	0.20 ± 0.01a	0.35 ± 0.02b	0.33 ± 0.07b	0.28 ± 0.00a
**Volatile phenols**								
m-Cresol	MS, LRI	1143	0.45 ± 0.01a	0.46 ± 0.01a	4.03 ± 0.56c	2.70 ± 0.42b	6.07 ± 0.34d	7.95 ± 0.61e
Phenol	MS, LRI	2012	1.68 ± 0.16d	1.57 ± 0.43cd	0.97 ± 0.15ab	1.50 ± 0.25bcd	0.89 ± 0.03ab	0.84 ± 0.09a
Ethylguaiacol	MS, LRI	2038	0.81 ± 0.08a	0.85 ± 0.13a	1.59 ± 0.11b	37.73 ± 2.37d	35.56 ± 2.28d	9.27 ± 0.71c
4-Diacetylaminophenol	MS, LRI	2084	0.00 ± 0.00a	0.00 ± 0.00a	0.00 ± 0.00a	0.47 ± 0.05b	0.1 ± 0.00c	0.00 ± 0.00a
p-Cresol	MS, LRI	2095	2.30 ± 0.09b	2.23 ± 0.11b	0.20 ± 0.03a	0.13 ± 0.02a	0.16 ± 0.03a	0.15 ± 0.02a
4-Ethylphenol	MS, LRI	2081	0.00 ± 0.00a	0.00 ± 0.00a	6.83 ± 0.89b	206.65 ± 41.09e	163.31 ± 11.53cd	155.07 ± 15.95c
2,4-Di-tert-butylphenol	MS, LRI	2305	0.00 ± 0.00a	0.00 ± 0.00a	2.61 ± 0.56c	2.78 ± 0.48c	1.48 ± 0.19b	2.28 ± 0.19bc

Note: UF1 and UF2: Unfermented SCG hydrolysates at day 0 and day14; LT: mono-inoculation of *L. thermotolerans* Concerto, LP: mono-inoculation of *L. plantarum* ML Prime, LT+Co-LP: co-inoculation of *L. thermotolerans* Concerto and *L. plantarum* ML Prime; LT+Se-LP: sequential inoculation of *L. thermotolerans* Concerto and *L. plantarum* ML Prime at day 4. LRI: linear retention index; determined on a DB-FFAF column relative to C7-C40 hydrocarbons. ANOVA statistical analysis of mean values (n=3) with different letters (a-e) at $p < 0.05$.

Table S3. Compound names related to numbers used in PCA analysis.

Number	Compound	Number	Compound	Number	Compound
1	a-Pyrone-6-carboxylic acid	26	Benzaldehyde	51	2-Vinylfuran
2	Propanoic acid	27	5-Methylfurfural	52	2,3-Pentanedione
3	Butanoic acid	28	Benzeneacetaldehyde	53	2-Heptanone
4	2-Propenoic acid	29	2,5-Dimethylbenzaldehyde	54	3-Octanone
5	Pentanoic acid	30	1-Methylpyrrole-2-carboxaldehyde	55	Heptenone
6	3-Methylbutanoic acid	31	Ethyl acetate	56	Acetoin
7	2-Butenoic acid	32	Vinyl acetate	57	Methyl 3-furoate
8	Hexenoic acid	33	*cis*-3-Hexenyl phenylacetate	58	2,6-Dimethyl pyrazine
9	Octanoic acid	34	Ethyl hexanoate	59	2,6-Methylethylpyrazine
10	Nonanoic acid	35	Ethyl hexenoate	60	Pyridazine
11	n-Decanoic acid	36	Methyl acetate	61	2-Vinylpyrazine
12	Benzoic acid	37	Ethyl heptanoate	62	Pyridine
13	3-Buten-2-ol	38	Isopropyl L-lactate	63	2-Acetylpyrrole
14	1-Pentanol	39	Ethyl sorbate	64	Linalool
15	1-Hexen-3-ol	40	Ethyl 4-hydroxyphenylacetate	65	*a*-Terpineol
16	2-Heptanol	41	Ethyl nonanoate	66	*m*-Cresol
17	3-Furylmethanol	42	Furfuryl acetate	67	Phenol
18	*cis*-Geraniol	43	Ethyl decanoate	68	*p*-Ethylguaiacol
19	Benzyl alcohol	44	Ethyl (*Z*)-4-decenoate	69	4-Diacetylaminophenol
20	2-Phenylethyl alcohol	45	Isopropyl acetate	70	*p*-Cresol
21	2-Ethyl-2-heptanol	46	2-Phenethyl acetate	71	4-Ethylphenol
22	3-Methyl pentanal	47	2,5-Dimethylfuran	72	2,4-Di-tert-butylphenol
23	2-Hexenal	48	2-Pentylfuran	73	Acetic acid
24	*trans*-Linalool oxide	49	2-Acetylfuran	74	Ethanol
25	Furfural	50	2-Acetyl-5-methylfuran		
